# Effect of *Mycoplasma hominis* and cytomegalovirus infection on pregnancy outcome: A prospective study of 200 Mongolian women and their newborns

**DOI:** 10.1371/journal.pone.0173283

**Published:** 2017-03-03

**Authors:** Byambaa Otgonjargala, Kathrin Becker, Gunchin Batbaatar, Sandag Tsogtsaikhan, Jamsranjav Enkhtsetseg, Altangerel Enkhjargal, Klaus Pfeffer, Ortwin Adams, Chimeddorj Battogtokh, Birgit Henrich

**Affiliations:** 1 Department of Microbiology and Immunology, School of Pharmacy and Biomedicine, Mongolian National University of Medical Sciences, Ulaanbaatar, Mongolia; 2 Department of Orthodontics, University Clinic, Düsseldorf, Germany; 3 Department of Obstetrics and Gynecology, School of Medicine, Mongolian National University of Medical Sciences, Ulaanbaatar, Mongolia; 4 Center for Environmental Health and Toxicology, Public Health Institute, Ulaanbaatar, Mongolia; 5 Institute of Medical Microbiology and Hospital Hygiene, Heinrich-Heine-University, Düsseldorf, Germany; 6 Institute of Virology, Heinrich-Heine-University, Düsseldorf, Germany; Miami University, UNITED STATES

## Abstract

In Mongolia, diagnostic tests for the detection of the sexually transmitted mycoplasmas, ureaplasmas, Herpes simplex virus (HSV), and cytomegalovirus (CMV) are currently not routinely used in clinical settings and the frequency of these STIs are enigmatic. The prevalence of these STI pathogens were prospectively evaluated among 200 Mongolian pregnant women and their newborns and correlated with pregnancy outcome. TaqMan PCRs were used to detect bacterial and viral STI pathogens in pre-birth vaginal swabs of the pregnant women and in oral swabs of their newborns. A standardized questionnaire concerning former and present pregnancies was developed and linear regression analysis was used to correlate pathogen detection with pregnancy outcome. Ureaplasmas were the most prevalent of the tested pathogens (positive in 90.5% positive women and 47.5% newborns), followed by mycoplasmas (32.5% and 7.5%), chlamydia (14.5% and 7.5%), trichomonas (8.5% and 4.0%) and gonococcus (0.5% and 0%). CMV was found in 46.5% of the pregnant women and in 10.5% of their newborns, whereas HSV-2 was detected in only two mothers. Multiple regression analyses indicate that colonization of the mothers with *U*. *urealyticum*, *M*. *hominis*, *T*. *vaginalis* or CMV is associated with transmission to newborns and that transmission of *M*. *hominis* or CMV from Mongolian pregnant women to offspring is associated with reduced neonatal length and gestational age. Thus, diagnostic tests for their detection should be implemented in the clinical settings in Mongolia.

## Introduction

Sexually transmitted infections (STI) constitute a major public health care problem worldwide, particularly among women and neonates, as they cause a significant socio-economic burden in both developed and developing countries [1]. Maternal genital tract infections are associated with complications such as ascending chorioamnionitis, premature rupture of membranes, preterm birth, miscarriage and low birth weight and neonatal length. Pathogens associated with these complications include *Chlamydia trachomatis*, *Neisseria gonorrhoeae*, *Mycoplasma hominis*, *Ureaplasma parvum* and *U*. *urealyticum*.

*M*. *hominis* and *Ureaplasma* spp. also reside as commensals in the human urogenital tract of 30–80% women, thus casting their pathogenic potential in case of detection into doubt. These organisms have low virulence and may reside in the uterus for months before transmission to the fetus and precipitating preterm labor [2]. Although known as causes of neonatal infections, they are unfortunately often undiagnosed because of low awareness or lack of diagnostic facilities [3].

Viral infections with herpes simplex virus type 2 (HSV-2) [4], or cytomegalovirus (CMV) may cause neonatal infection, which can vary after intrauterine transmission from mild to severe including intrauterine growth retardation, low birth weight and CNS disorders [5], both of which are also significant health care issues [6].

In Mongolia, STI are common and *N*. *gonorrhoeae*, *C*. *trachomatis*, human papilloma virus or *Trichomonas vaginalis* were found in 53% of female attendees of a Mongolian STD clinic [7]. 14,904 cases of STI were registered in 2014, taking up 39.9% of communicable diseases, and compared to 2013, increased by 1.5 per 10.000 population or 414 cases. 32.5% of STI were gonorrhea, 41.9% syphilis, 25.4% trichomoniasis and 0.2% HIV. Of the examinations done on 84,399 pregnant women, 1600 cases (1.9%) of syphilis, 538 cases (0.7%) of gonorrhea, and 1425 cases (1.9%) of trichomoniasis were detected in 2014 [8]. However, data on the prevalence of urogenital mycoplasma and ureaplasma are still not available in Mongolia.

Rapid and reliable tests are required for early detection and treatment of STI to prevent adverse pregnancy outcomes and neonatal infections. Multiplex PCRs were designed for the simultaneous detection of multiple microbial pathogens in the urogenital tract [9, 10]. These molecular assays have been increasingly used for the detection of common etiologic agents in genital discharge and pregnancy, but in Mongolia, they are currently not routinely used in clinical settings.

In this study, a set of TaqMan PCR assays was employed for the quantitative detection of *C*. *trachomatis* (Ct) and *N*. *gonorrhoeae* (Ng), *M*. *genitalium* (Mg), *M*. *hominis* (Mh), *U*.*urealyticum* (Uu), *U*.*parvum* (Up), *Trichomonas vaginalis* (Tv), CMV and HSV-2 to analyze the incidence of these pathogens in 200 Mongolian pregnant women and their newborns and to correlate pathogen detection with pregnancy outcome.

## Materials and methods

### Study population

The cross sectional study was carried out on 200 healthy pregnant women and their newborns from the First Maternity Hospital, Second Maternity Hospital, Third Maternity Hospital and National Center for Maternal and Child Health of Mongolia. Samples were collected during a period of 3 months from July to September 2014. The pregnant women provided written informed consent to participate with their newborns in this study. The study protocol was approved by the Medical Ethics Committee, Mongolian National University of Medical Sciences (14-15/1A).

### Specimen collection

Vaginal secretions of the pregnant women were collected from the mid-vaginal wall between 1 and 24 hours pre-birth by polystyrene swab. Oral swabs were taken from newborns, all of which were delivered vaginally, within 15 minutes of birth. One swab per patient was taken by a physician. Specimens were transported to the microbiological laboratory of MNUMS in PBS-buffer (phosphate buffer saline solution).

### Questionnaire

A questionnaire was filled up for each woman to obtain general information on the history, outcome and related risk factors of previous and the present pregnancy. Gestational age was calculated on basis of last menstrual period and day of birth.

### DNA extraction method

DNA was extracted from the vaginal and oral swabs by using Blood & Cell Culture DNA Midi Kit (Qiagen, Hilden Germany) at the microbiological laboratory of MNUMS in Mongolia. The DNA solution was dried over night at 80°C Celsius and sent to the Institute of Medical Microbiology and Hospital Hygiene at the Heinrich-Heine-University of Düsseldorf, Germany, where it was resolved in 100μl aqua dest. and stored at -20°C until use in Real time PCR.

### TaqMan PCR assays

TaqMan PCRs for the detection of *C*. *trachomatis*, *N*. *gonorrhoeae*, *Ureaplasma* spp. and *Mycoplasma* spp. [9] and *Trichomonas vaginalis* [11], with detection limits of 25 genome copies per reaction, were carried out on a CFX96 Real time PCR machine (Biorad, Munich, Germany) in a total volume of 25μl containing 2.5μl DNA-solution of the swab specimens in NoROX PCR Mastermix (Qiagen, Hilden, Germany) with primers and probes concentrations as published. Fifty copies of an internal control (IC) plasmid were added as an inhibition control [9]. Thermal cycling conditions were as follows: 10min at 95°C for initial denaturation followed by 45 cycles of 95°C for 15s and 60°C for 1min. HSV-2- and CMV-PCRs with detection limits each of 5 copies per reaction were performed with an ABI 7500 sequence detector system (Applied Biosystems, Darmstadt, Germany) as described before [10]. BlastN search of primers and probes showed no homology with other known STI pathogens, and clinical samples positive for other human herpesviruses and STI pathogens no cross reaction, too.

### Statistical analysis

The statistical analysis was conducted using the software program RStudio, version 0.99.896 [12]. Prior to the analyses, the absolute numbers of pathogens were normalized by computing the pathogen load to: 1. human cells (GAPDH-genome equivalents); 2. total sample DNA or 3. total bacterial DNA (16S rDNA-copies/sample). Multiple regression analysis was used to assess the association between pathogen loads of mothers or newborns and the outcome variables (gestational age, neonatal length, weight, head circumferences and bacterial vaginosis). For this analysis, normal distribution of the residuals and homogeneity of variance were verified, and leverage plots were analyzed to identify potential outliers that may distort the analyses. Cook’s distance, which represents the scaled change in fitted values, was calculated to identify data points with large residuals or high leverage that might distort the statistical analysis. Additionally, absence of multi-collinearity among the pathogen species was verified for both groups, i.e. mothers and offspring, by computing variance inflation factors for the standard errors of linear model coefficient estimates (DAAG package).

To assess vertical pathogen transfer between mothers and offspring, the correlation of respective pathogen loads was assessed by computing Spearman Rho (only pathogens with mean maternal load > .0005 were included in the analysis). Spearman’s rank correlation was also used to assess correlation between maternal age and pregnancy outcome. Graph Pad Prism software (Vers. 5.01, GraphPad Software, San Diego, CA) was used for design of column scatter plots. Results were given as means ± standard deviation (SD). Kruskal-Wallis rank sum test was used to calculate statistically significant differences (p-values < 0,05) between GA-groups, outcome of present pregnancy or maternal Up, Mh or CMV and parity, maternal or neonatal Up, Mh and CMV and apgar-score, and maternal GAPDH or sample DNA and level of leukocytosis in vaginal smear test. Differences between pregnancy outcome and neonatal gender were assessed using the Wilcoxon rank sum test. Results were found significant at alpha = 0.05.

## Results

A cohort of 200 Mongolian pregnant women, aged between 17 and 41 years, and their newborn babies, born between 35 to 40 weeks of gestation, was studied for presence of STI pathogens. A standardized questionnaire was developed to collect data concerning the outcome of the present and, if applicable, former pregnancies. 64.5% of the women were married, 48.5% had a higher education level (secondary education or college degree) and 78% of women had no history of STI (Table 1). Previous STI was ascertained in a face-to-face interview of 71 pregnant women and taken from written medical history of 129 women. In the present pregnancies syphilis (0.5%) was less frequently detected than bacterial vaginosis (19%) or the presence of candida (27%).

**Table 1:** Characteristics of 200 pregnant women from Mongolia, Ulaanbaatar.

Variables	n	%
**Maternal hospital**		
First maternity hospital	50	25.0
Second maternity hospital	50	25.0
Third maternity hospital	50	25.0
National Center for Maternal and Child Health hospital	50	25.0
**Age**		
≥31 years old	44	22.0
21–30 years old	129	64.5
≤20 years old	27	13.5
**Marital status**		
Married	129	64.5
Single	62	31.0
Divorced	9	4.5
**Education level**		
Basic education	6	3.0
Secondary education	97	48.5
Specialized secondary education /college	36	18.0
Higher education	61	30.5
**History of STI**		
Yes	44	22.0
No	156	78.0
Nugent score for smear test of present pregnancy*		
Normal	96	48.0
Intermediate	65	32.5
Bacterial vaginosis	38	19.0
Unknown	1	0.5
**Leukocyte for smear test of present pregnancy**		
Rare or not (< 5 leukocytes/microscopic field)	85	42.5
Medium (5–25 leukocytes/microscopic field)	84	42.0
High (> 25 leukocytes/microscopic field)	30	15.0
Unknown	1	0.5
**Candida detection of present pregnancy**		
Yes	54	27.0
No	144	72.0
Unknown	2	1.0
**Syphilis of present pregnancy**		
Yes (TPHA-positive)	1	0.5
No (TPHA-negative)	199	99.5

* detailed information of Nugent scoring is given in the legend of S1 Table

**Fig 1:**
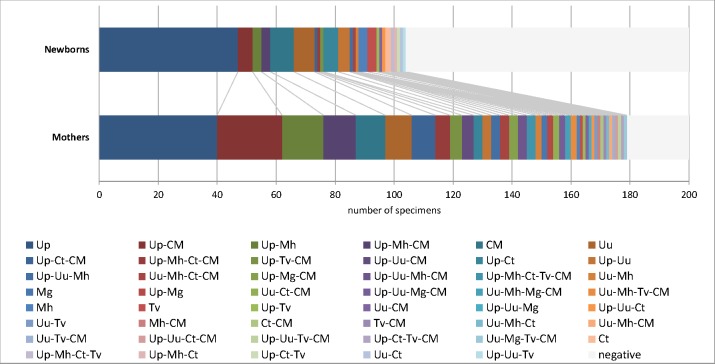
Co-localizing STI pathogens. Stacked bar charts indicate the decreasing number of specimens (with respect to the maternal colonization; from left) with detected pathogens and pathogen-communities in the 200 maternal vaginal specimens (Mothers) and 200 neonatal oral specimens (Newborns). Abbreviations indicate the respective microorganisms: Up, *U*. *parvum*, Uu, *U*. *urealyticum*; Mh, *M*. *hominis*; Mg, *M*. *genitalium*; Ct, *C*. *trachomatis*, Tv, *T*.*vaginalis*; and CM, CMV.

TaqMan PCR was used to detect and quantify the STI organisms in pre-birth vaginal samples of the women and in oral samples of the neonates. No pathogen was detected in only 10.5% of the pregnant women (n = 21/200) and 48% of their newborns (96/200) (Fig 1). With regard to the neonates, *N*. *gonorrhoeae*, *M*. *genitalium* and HSV-2 were not found in the oral swabs. Ureaplasmas were the most prevalent pathogens in pregnant women (90.5%) and in their newborn babies (47.5%) with *U*. *parvum* representing the dominant species (68% (n = 136) and 39% (n = 78) in mother and child respectively). This was followed by mycoplasmas (32.5% / 7.5%), *M*. *hominis* (26% / 7.5%), *C*. *trachomatis* (14.5% / 7.5%), *T*. *vaginalis* (8.5% / 4.0%) and *N*. *gonorrhoeae* (0.5% / 0%) in mother and child respectively. Viruses tested were detected in 47.5% of the pregnant women (46.5% CMV and 1% HSV-2) and in 10.5% of the neonates (solely CMV).

Colonization with only one of the pathogens tested was found in 33% maternal and 37% neonatal specimens; here too a predominance of *U*. *parvum*. Although multiple regression analysis of data did not point to a statistical evidence for a co-linearity in between distinct members of the pathogens tested, ureaplasmas were most often found in the maternal (35.5%) and neonatal specimens (7%) in combination with mycoplasmas (in 22 maternal and 5 neonatal specimens), CMV (in 27 maternal and 6 neonatal specimens) or both pathogens (in 22 and 3 specimens, respectively). The more rarely detected ureaplasma species, *U*. *urealyticum*, *M*. *hominis* and CMV were found more often in combination with each other in mothers (18.5%; n = 37/200) than alone. This polymicrobial combination was less prominent in newborns (4%; n = 8/200), where colonization with only one of these pathogens dominated. On the other hand, some combinations of pathogens were detected in neither maternal nor in neonatal specimens. *M*. *hominis*, *T*. *vaginalis* and *C*. *trachomatis* were never found complexed in different combinations with each other in ureaplasma-free specimens.

### Outcome of vertical transmission from mothers to neonates

**Fig 2:**
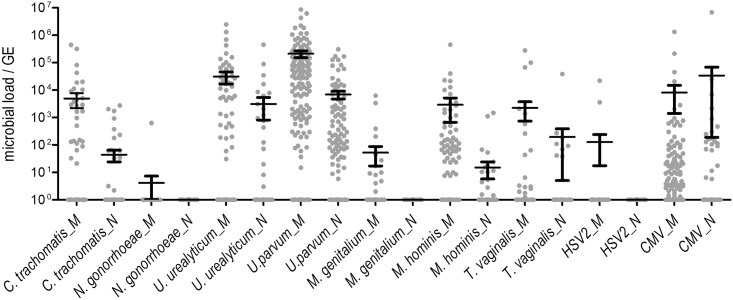
STI-pathogen load in mothers and their newborns. Quantities of each pathogen were normalized to human cells (pathogen-GE /10^6^ GAPDH-GE) and the mean (+/- standard deviation) of the 200 values of mothers (_M) and newborns (_N) depicted as (x+1) to visualize even the negative detections in column scatter plot.

The pathogen load of each sample, quantified by TaqMan PCR, was normalized to human cells (genome equivalents (GE) of human GAPDH), sample DNA content or total bacterial load (16S rDNA copies/ sample) and averaged for each species in maternal and neonatal samples to eliminate variations of sampling (S1 Table). As shown in Fig 2, the microbial load of *U*. *parvum* in maternal samples (2,10E+05 (+/-) 8,43E+05) was higher than that of *U*. *urealyticum* (3,09E+04 (+/-) 2,06E+05) or CMV (8,10E+03 (+/-) 9,47E+04). In oral samples of neonates, quantities of CMV (3,38E+04 (+/-) 4,75E+05) were greater than those of *U*. *parvum* (6,88E+03 (+/-) 3,12E+04) and *U*. *urealyticum* (3,09E+03 (+/-) 3,23E+04) and were lowest for *M*. *hominis* (1,50E+01 (+/-) 1,30E+02).

When assessing correlations between pathogen loads in mothers and offspring, which were based on GAPDH-normalization, a significant correlation between all species with mean load >.0005 was detected. These findings provide evidence that a maternal colonization with *U*. *urealyticum*, *M*. *hominis*, *T*. *vaginalis* or detection of CMV was associated with a significantly higher risk of transmission to the fetus. No cross-correlation was detected among the pathogen species of both groups, i.e. mothers and offspring.

**Fig 3:**
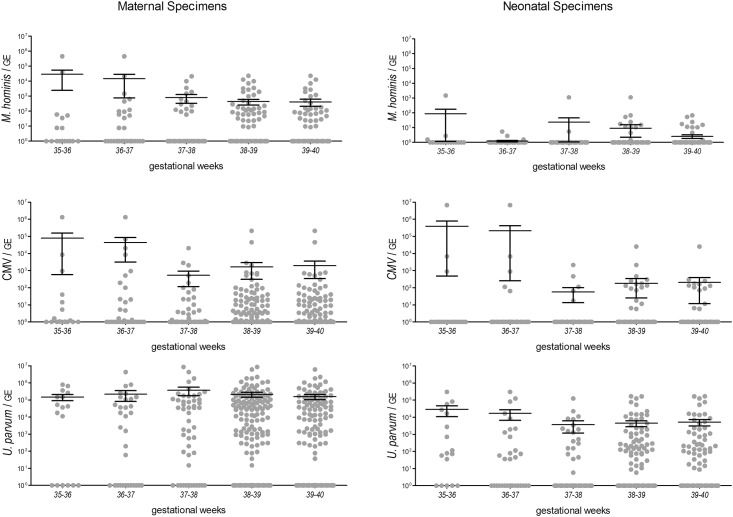
Quantities of *M*. *hominis*, CMV and *U*. *parvum* with respect to gestational age. Column scatter plots indicate the mean genome equivalents of *M*. *hominis*, CMV and *U*. *parvum* in maternal and neonatal specimens, normalized to 10^6^ GAPDH genome equivalents, in 2 GA -weeks intervals from 35–36 to 39–40. Statistically significant differences were assessed by Kruskal-Wallis test between GA-groups 35–36 and 38–39 or 39–40, respectively (p-values < 0,05).

To assess linear associations between the quantities of STI pathogens tested and outcome variables, multiple regression models were employed. Leverage plots pointed at two potential outliers. When evaluating regression analyses, large cook distances were found for two of the maternal subjects and one of the newborns. Thus outcomes from PCR were verified and it was tested if the association still exists if these two observations were removed. When including all values, multiple regression analyses yielded a significant model (Table 2). Mh and CMV were found to be significant in both groups, whereas Up was found to be significant only in the neonatal group; and that only after GAPDH- or DNA-normalization of the pathogen load (S2 Table). Once the two observations with large cook distances were excluded from the analyses, CMV failed significance in both groups, whereas Mh remained significant in both groups and Up remained significant in the offspring group. However, re-evaluation of PCR outcomes verified plausibility of the outcomes in question. As gestational age (GA) may have a 2-week uncertainty due to an unknown conception, the mean maternal and neonatal CMV, *M*. *hominis* and *U*. *parvum* quantities were calculated with respect to a 2-weeks gestational age interval (Fig 3). Even under this condition, the mean microbial load of all pathogen groups, except for maternal Up, declined with increasing gestational age.

**Table 2:** Correlation of pathogen load and pregnancy outcome.

**neonatal length / pathogen load**^1^						**[neonatal length/maternal length] / pathogen load**					
Coefficients:	Estimate	Std. Error	t value	Pr(>|t|)	Sign.	Coefficients:	Estimate	Std. Error	t value	Pr(>|t|)	Sign.
(Intercept)	5,03E+01	2,09E-01	240,461	< 2,00E-16	***	(Intercept)	0,308247	0,001416	217,761	< 2,00e-16	***
CtM	1,77E+00	5,09E+00	0,347	0,72886		CtM	0,020805	0,034458	0,604	0,54671	
UuM	-1,24E+00	1,00E+00	-1,24	0,21644		UuM	-0,004092	0,006763	-0,605	0,54587	
UpM	1,50E-01	2,44E-01	0,615	0,53961		UpM	0,002714	0,001652	1,642	0,10213	
MgM	-8,30E-01	3,94E+02	-0,002	0,99832		MgM	0,849546	2,665027	0,319	0,75024	
MhM	-2,65E+01	6,23E+00	-4,257	3,25E-05	***	MhM	-0,167668	0,042148	-3,978	9,86E-05	***
TvM	6,81E+00	9,80E+00	0,696	0,48758		TvM	0,081669	0,066269	1,232	0,21932	
HSVM	1,58E+01	1,33E+02	0,119	0,90573		HSVM	-0,263227	0,902551	-0,292	0,77087	
CMM	-6,15E+00	2,09E+00	-2,945	0,00364	**	CMM	-0,046355	0,014119	-3,283	0,00122	**
Residual standard error: 2,788 on 191 degrees of freedom						Residual standard error: 0,01886 on 191 degrees of freedom					
Multiple R-squared: 0,1306; Adjusted R-squared: 0,09418						Multiple R-squared: 0,1385; Adjusted R-squared: 0,1024					
F-statistic: 3,586 on 8 and 191 DF, p-value: 0,000668						F-statistic: 3,839 on 8 and 191 DF, p-value: 0,0003275					
(Intercept)	5,03E+01	2,08E-01	241,566	< 2,00E-16	***	(Intercept)	0,30865	0,001408	219,248	< 2,00E-16	***
CtB	2,22E+02	7,48E+02	0,297	0,76649		CtB	1,658472	5,058787	0,328	0,74339	
UuB	-6,99E+00	6,18E+00	-1,13	0,25989		UuB	-0,035121	0,041832	-0,84	0,40219	
UpB	7,76E+00	6,69E+00	1,16	0,24743		UpB	0,026599	0,045238	0,588	0,55723	
MgB	NA	NA	NA	NA		MgB	NA	NA	NA	NA	
MhB	-4,90E+03	1,54E+03	-3,19	0,00166	**	MhB	-33,662343	10,394743	-3,238	0,00142	**
TvB	4,48E+01	7,38E+01	0,607	0,54442		TvB	0,587342	0,499502	1,176	0,2411	
HSVB	NA	NA	NA	NA		HSVB	NA	NA	NA	NA	
CMB	-1,23E+00	4,21E-01	-2,93	0,0038	**	CMB	-0,009383	0,002844	-3,299	0,00116	**
Residual standard error: 2,818 on 193 degrees of freedom						Residual standard error: 0,01906 on 193 degrees of freedom					
Multiple R-squared: 0,1022; Adjusted R-squared: 0,07434						Multiple R-squared: 0,1104; Adjusted R-squared: 0,08273					
F-statistic: 3,663 on 6 and 193 DF, p-value: 0,001812						F-statistic: 3,991 on 6 and 193 DF, p-value: 0,0008645					
**gestational age / pathogen load**						**neonatal weight / pathogen load**					
Coefficients:	Estimate	Std. Error	t value	Pr(>|t|)	Sign.	Coefficients:	Estimate	Std. Error	t value	Pr(>|t|)	Sign.
Intercept)	3,85E+01	7,44E-02	516,928	< 2,00E-16	***	(Intercept)	3,13451	0,04149	75,557	< 2,00E-16	***
CtM	-1,14E+00	1,81E+00	-0,631	0,529		CtM	0,20425	1,00987	0,202	0,84	
UuM	-3,46E-01	3,55E-01	-0,974	0,331		UuM	-0,25656	0,19821	-1,294	0,197	
UpM	-1,24E-02	8,69E-02	-0,143	0,887		UpM	0,04477	0,04842	0,925	0,356	
MgM	3,86E+01	1,40E+02	0,275	0,783		MgM	33,73993	78,10488	0,432	0,666	
MhM	-6,15E+00	2,22E+00	-2,775	0,006	**	MhM	-2,03097	1,23525	-1,644	0,102	
TvM	2,03E+00	3,48E+00	0,584	0,560		TvM	1,92647	1,94217	0,992	0,322	
HSVM	-2,20E+00	4,74E+01	-0,046	0,963		HSVM	14,42091	26,45137	0,545	0,586	
CMM	-1,75E+00	7,42E-01	-2,362	0,019	*	CMM	-0,18347	0,41379	-0,443	0,658	
Residual standard error: 0,9911 on 191 degrees of freedom						Residual standard error: 0,5527 on 191 degrees of freedom					
Multiple R-squared: 0,07369; Adjusted R-squared: 0,03489						Multiple R-squared: 0,0343; Adjusted R-squared: -0,006144					
F-statistic: 1,899 on 8 and 191 DF, p-value: 0,06218						F-statistic: 0,8481 on 8 and 191 DF, p-value: 0,5615					
(Intercept)	3,85E+01	7,10E-02	541,903	< 2,00E-16	***	(Intercept)	3,13971	0,04075	77,052	< 2,00E-16	***
CtB	4,53E+02	2,55E+02	1,777	0,07713	,	CtB	118,91499	146,4279	0,812	0,418	
UuB	-1,57E+00	2,11E+00	-0,745	0,45742		UuB	-1,58261	1,21084	-1,307	0,193	
UpB	-6,68E+00	2,28E+00	-2,928	0,00383	**	UpB	0,32917	1,30944	0,251	0,802	
MgB	NA	NA	NA	NA		MgB	NA	NA	NA	NA	
MhB	-1,55E+03	5,24E+02	-2,957	0,0035	**	MhB	-411,77598	300,87854	-1,369	0,173	
TvB	1,31E+01	2,52E+01	0,519	0,60417		TvB	11,80074	14,45822	0,816	0,415	
HSVB	NA	NA	NA	NA		HSVB	NA	NA	NA	NA	
CMB	-3,68E-01	1,44E-01	-2,567	0,011	*	CMB	-0,03594	0,08233	-0,437	0,663	
Residual standard error: 0,9615 on 193 degrees of freedom						Residual standard error: 0,5518 on 193 degrees of freedom					
Multiple R-squared: 0,1191; Adjusted R-squared: 0,09171						Multiple R-squared: 0,02724; Adjusted R-squared: -0,002997					
F-statistic: 4,349 on 6 and 193 DF, p-value: 0,0003839						F-statistic: 0,9009 on 6 and 193 DF, p-value: 0,4953					
**neonatal head circumference / pathogen load**						**bacterial vaginosis / pathogen load**					
Coefficients:	Estimate	Std. Error	t value	Pr(>|t|)	Sign.	Coefficients:	Estimate	Std. Error	t value	Pr(>|t|)	Sign.
(Intercept)	34,9841	0,05791	604,122	<2,00e-16	***	(Intercept)	6,99E-01	5,90E-02	11,846	<2,00E-16	***
CtM	1,37487	1,39009	0,989	0,324		CtM	3,80E-01	1,44E+00	0,264	0,792	
UuM	0,22253	0,27333	0,814	0,417		UuM	3,85E-01	2,82E-01	1,365	0,174	
UpM	0,04025	0,06777	0,594	0,553		UpM	5,25E-02	6,89E-02	0,762	0,447	
MgM	49,06387	107,50272	0,456	0,649		MgM	1,24E+02	1,11E+02	1,113	0,267	
MhM	-0,16423	1,70173	-0,097	0,923		MhM	-1,48E+00	1,76E+00	-0,84	0,402	
TvM	-2,49903	2,67295	-0,935	0,351		TvM	-2,23E+00	2,76E+00	-0,806	0,421	
HSVM	34,22078	36,40251	0,94	0,348		HSVM	-1,89E+01	3,76E+01	-0,503	0,615	
CMM	0,25516	0,56951	0,448	0,655		CMM	1,40E-01	5,89E-01	0,238	0,812	
Residual standard error: 0,7605 on 185 degrees of freedom						Residual standard error: 0,7865 on 191 degrees of freedom					
Multiple R-squared: 0,02192,Adjusted R-squared: -0,02038						Multiple R-squared: 0,03402; Adjusted R-squared: -0,006435					
F-statistic: 0,5182 on 8 and 185 DF, p-value: 0,8418						F-statistic: 0,841 on 8 and 191 DF, p-value: 0,5677					
(Intercept)	35,02586	0,05701	614,384	< 2,00E-16	***	(Intercept)	0,73903	0,05807	12,727	< 2,00E-16	***
CtB	-131,33501	210,69087	-0,623	0,534		CtB	-189,16839	208,66839	-0,907	0,366	
UuB	-0,71029	1,66603	-0,426	0,67		UuB	1,1265	1,72551	0,653	0,515	
UpB	-1,98771	2,56517	-0,775	0,439		UpB	-2,22579	1,86603	-1,193	0,234	
MgB	NA	NA	NA	NA		MgB	NA	NA	NA	NA	
MhB	186,00524	414,37007	0,449	0,654		MhB	232,99465	428,76964	0,543	0,587	
TvB	-13,63091	19,89219	-0,685	0,494		TvB	-18,9087	20,60382	-0,918	0,360	
HSVB	NA	NA	NA	NA		HSVB	NA	NA	NA	NA	
CMB	0,07135	0,11328	0,63	0,53		CMB	0,03917	0,11733	0,334	0,739	
Residual standard error: 0,7591 on 187 degrees of freedom						Residual standard error: 0,7863 on 193 degrees of freedom					
Multiple R-squared: 0,01508; Adjusted R-squared: -0,01652						Multiple R-squared: 0,0244; Adjusted R-squared: -0,00593					
F-statistic: 0,4773 on 6 and 187 DF, p-value: 0,8247						F-statistic: 0,8045 on 6 and 193 DF, p-value: 0,5675					

^1^ Pathogen load normalized to human GAPDH-genome equivalents;

^2^ Significance codes (Sign.): 0 '***' 0,001 '**' 0,01 '*' 0,05 ',' 0,1 ' ' 1;

M, _mothers; _B, _babies; Ct, C. trachomatis; Uu, *U*. *urealyticum*; Up, *U*. *parvum*; Mg, *M*. *genitalium*; Mh, *M*. *hominis*; Tv, *T*. *vaginalis*; HSV, HSV-2; CM, CMV;

For the outcome variable neonatal length, Mh and CMV were found to have significant influence, independent on the kind of pathogen normalization (S2 Table) and a normalization of neonatal length to maternal length (Table 2). Since even the level of leukocytosis did not significantly influence the quantity of human cells (GAPDH-GE) (Table 3), further statistical analyses were based on GAPDH-normalization only.

**Table 3:** Correlation of pregnancy outcome variables.

Correlation method	Variables	Correlation parameters	p-value	Sign.
Pearson's product-moment correlation	neonatal length, neonatal weight	t = 12,1852, df = 198	< 2,20E-16	***
Spearman's rank correlation	maternal / neonatal *T*. *vaginalis*	rho = 0,58	< 0,0001	***
	maternal / neonatal *U*.*urealyticum*	rho = 0,57	< 0,0001	***
	maternal / neonatal *M*. *hominis*	rho = 0,48	< 0,0001	***
	maternal / neonatal CMV	rho = 0,42	< 0,0001	***
	neonatal length, maternal age	rho = 0,10	0,1687	
	neonatal weight, maternal age	rho = 0,19	6,09E-03	*
	gestational age, maternal age	rho = -0,01	0,8690	
	bacterial vaginosis, leukocytosis	Rho = 0.41	1.591e-09	***
Kruskal-Wallis rank sum test	neonatal length, parity	chi-squared = 11,8955, df = 2	2;61E-03	**
	neonatal weight, parity	chi-squared = 11,2019, df = 2	3,69E-03	**
	gestational age, parity	chi-squared = 18,9418, df = 2	7,71E-05	***
	maternal *U*. *parvum*, parity	chi-squared = 2,562, df = 2	0,2778	
	maternal *M*. *hominis*, parity	chi-squared = 3,2726, df = 2	0,1947	
	maternal CMV, parity	chi-squared = 0,7873, df = 2	0,6746	
	maternal *U*. *parvum*, apgar-score	chi-squared = 2,9226, df = 3	0,4037	
	maternal *M*. *hominis*, apgar-score	chi-squared = 4,.0072, df = 3	0,2607	
	maternal CMV, apgar-score	chi-squared = 3.3349, df = 3	0,3428	
	neonatal *U*. *parvum*, apgar-score	chi-squared = 1,0696, df = 3	0,7844	
	neonatal *M*. *hominis*, apgar-score	chi-squared = 4,9501, df = 3	0,1755	
	neonatal CMV, apgar-score	chi-squared = 5,155, df = 3	0,1608	
	maternal GAPDH, leukocytosis	chi-squared = 0,2614, df = 2	0,8775	
	maternal DNA, leukocytosis	chi-squared = 1,0703, df = 2	0,5856	
Wilcoxon rank sum test	neonatal length, gender	W = 4664,5	0,4486	
	neonatal weight, gender	W = 4584,5	0,3463	
	gestational age, gender	W = 4783,5	0,5950	

Signif. codes: 0 '***' 0.001 '**' 0.01 '*' 0.05 '.' 0.1 ' ' 1

When evaluating the outcome variable neonatal weight, multiple regression analyses failed to produce statistical significance (Table 2). However, the reduction of neonatal length correlated with a reduction of neonatal weight (Table 3).

No statistically significant correlation was detected between 1. pathogen load and neonatal head circumferences (Table 2), 2. pregnancy outcome (e.g. neonatal length, weight or gestational age) and neonatal gender, 3. maternal Up-, Mh- or CMV- load and apgar-score or parity, and 4. bacterial vaginosis and pathogen load or pregnancy outcome (neonatal length, weight or gestational age) (Table 3). Interestingly, neonatal weight significantly decreased with higher maternal age, and neonatal weight, length or gestational age decreased with higher parity (Table 3).

## Discussion

In pregnancy the maternal immune system changes to facilitate tolerance to fetal antigens [13]. This allows a higher susceptibility to infections, caused by less-pathogenic microorganisms such as ureaplasma and mycoplasma, affecting maternal and neonatal complications in pregnancy [14]. Additionally, human cytomegalovirus (CMV) is a leading cause of congenital infections worldwide, but reliable data of prevalence and outcome from developing countries are not available so far.

Prevalence and epidemiology of urogenital mycoplasma and ureaplasma, especially in pregnancy, are still unknown in Mongolia. In the present study a high prevalence of *U*. *parvum* (68.0%/39.5%) was detected in 200 mothers and their newborns. Multiple regression analysis revealed a vertical transmission of *U*. *urealyticum*, *T*. *vaginalis*, *M*. *hominis* and CMV, but not of *U*. *parvum*, from mothers to offspring. This is in good accordance with the finding that *U*. *parvum* was often found in vaginal samples of pregnant women in high concentrations, even in cases in which neonatal outcome was unaffected (personal observation). Interestingly, a statistically significant negative impact on gestational age due to neonatal colonization with *M*. *hominis*, or CMV or *U*. *parvum* was found. In a prospective study of 126 Greek women with preterm delivery and 125 women with full-term delivery, the vertical ureaplasma transmission to offspring was nearly twice the rate in preterm delivery (33%) than full-term delivery (17%) [15] underlining the impact of ureaplasmas on pregnancy outcome [16]. Colonisation of the urogenital tract also has other consequences. A study from Iran showed that the detection rate of *M*. *hominis* and/or *Ureaplasma* spp. was 2.4-fold higher in vaginal swabs of infertile women (n = 150) than of fertile women (n = 200) [17]. Olomu et al published in 2009 that the presence of ureaplasma in placental tissue before 28 gestational weeks was associated by a higher risk of preterm birth and neonatal complications such as intraventricular hemorrhage, inflammation and brain lesions [18]. Da Silva and colleagues screened 108 neonates with very low birth weight (VLBW) for ureaplasma and development of bronchopulmonary dysplasia (BPD) and did not find a significant correlation [19]. Unfortunately, in these studies, detection of ureaplasma was based on culture thus the lack of discrimination between *U*. *parvum* and *U*. *urealyticum* hindered the differentiation of the pathogenic potential of each species. In the study of Patel et al. colonization of the lower respiratory tract with genital mycoplasmas was analyzed at a species level in 319 pediatric patients, aged between 0.1 and 20 years: Highest detection rates of *U*. *urealyticum*, *M*. *hominis* and *M*. *genitalium* were detected in the age group 0–0.5 years and then declined, whereas *U*. *parvum* remained relatively constant over the age range of 0.1 to 20 years suggesting a less pathogenic behavior [20]. In a prospective study with 100 ventilated premature neonates, both ureaplasma species were detected separately by PCR and *U*. *urealyticum* was detected as a more important predictor for the development of a bronchopulmonary disease (BPD) than a decreased gestational age [21]. Our data suggest that neonatal colonization with *U*. *parvum*, but not *U*. *urealyticum* is associated with a reduced gestational age. This is in good accordance to the data of Prince et al., in which *U*.*parvum* was found to be associated with preterm delivery [22].

Clinical symptoms such as growth restriction have also been described in neonates with congenital CMV, although most of them are asymptomatic at birth [14]. CMV is highly under-recognized world-wide. In Australia, CMV was identified as the leading infectious cause of congenital malformation [23]. In addition, infections with CMV as well as *M*. *hominis* and *Ureaplasma* spp. bear an increased risk of miscarriage (spontaneous loss of pregnancy < 24 gestational weeks) [24, 25]. These results correspond to our findings that CMV and *M*. *hominis* are associated with preterm birth (< 37 gestational weeks) and a lower neonatal length. Our findings of a more frequent co-localization of *M*. *hominis* with *Ureaplasma* spp., although statistical not significant, was in good accordance with the study results of Kwak et al., which revealed that vaginal *M*. *hominis* tends to be detected together with *Ureaplasma* spp.. Patients carrying both bacterial genera had more severe adverse pregnancy outcomes than patients, who were positive for only ureaplasma [26]. McDonagh et al. studied the incidence of a similar spectrum of pathogens as we did (ureaplasma, mycoplasma, chlamydia, CMV and HSV-1 and -2) in biopsies of placenta and decidua from women with healthy pregnancies. The dominance of viruses in the decidua and of bacteria in the placenta suggested a different cell tropism and colonization behavior for CMV and bacteria [27]; in contrast to the polymicrobial community that we found in the maternal vaginal swabs. Their finding that *M*. *genitalium* infections did not appear to affect pregnancy outcome could not be confirmed by our results due to the low incidence of *M*. *genitalium* in the 200 Mongolian pregnant women tested and its absence in offspring.

Bacterial vaginosis (BV) is a syndrome which is characterized by a shift in the vaginal flora from a lactobacilli-dominated to a poly-microbial flora. BV is associated with a risk of respiratory distress and neonatal sepsis among full-term neonates [28] and, in presence of *M*. *hominis*, also associated with a risk of preterm birth [29]. In the present study, BV strongly correlated with vaginal leukocytosis, a constellation, which was postulated by Geisler et al as strong predictor of vaginal or cervical infections [30]. Although *M*. *hominis*, *Ureaplasma* spp. and CMV are found in the human microbiome during BV [31, 32], a statistically significant correlation between the pathogen load and BV or vaginal leukocytosis was not found in this study.

Infections with *C*. *trachomatis* or HSV are controversially discussed in the literature: some studies indicate an increased miscarriage risk, whereas others do not [25]. However, in the present study and in a former case-control study by Silveira et al. preterm birth (gestational age < 37 weeks) was found not to be associated with *C*. *trachomatis* detection [33].

The present study may have some limitations as the putative link of Mh, Up and CMV to neonatal length and premature labour is an interesting finding, but not statistically justified considering the small numbers of positive cases, the uncertainty of the gestational age (+/- 2 week) and exclusion of women with spontaneous abortion and of newborns with very low birth weight (<2.500g).

Further limitations of the study are the absent CMV seroprevalence data of the mothers. In developing nations with highly seropositive populations, high rates (1 to 5%) of neonatal infections have been reported [34]. The fact that CMV was found in 10.5% of the newborns in the present study suggests that the CMV seroprevalence in Mongolia is high. CMV infections of the newborn can result from primary infection in pregnancy or following reactivation of a previous infection, where more severe sequelae of the newborns are mostly associated with primary infections. In the present study no cases of severe sequelae of the newborns were reported and the association of CMV-infection and smaller neonatal length can be classified as a mild sequelae.

## Conclusions

In this study *Mycoplasma hominis*, cytomegalovirus and to some extent *U*. *parvum*, were shown to affect pregnancy outcome in the 200 Mongolian women studied. To prove this interesting finding for universal validity, molecular STI screening methods should be established in Mongolia, including *M*. *hominis*, *U*. *parvum* and CMV. As treatment of genital mycoplasmas in colonized pregnant women in late pregnancy is associated with a lower rate of premature labor and neonatal complications [35], preventive treatment should be considered to improve maternal and child health. STI detection, prevention and control strategies for pregnant women in Mongolia will likely help to prevent adverse pregnancy outcomes.

## Supporting information

S1 Table Pathogen load and outcome variables. (XLSX)

S2 Table Impact of pathogen load normalization in multiple regression analyses. (XLSX)
